# Comparison of the frequency of loss‐of‐function *LZTR1* variants between schwannomatosis patients and the general population

**DOI:** 10.1002/humu.24376

**Published:** 2022-04-14

**Authors:** Fanxuan Deng, D. Gareth Evans, Miriam J. Smith

**Affiliations:** ^1^ Division of Evolution, Infection and Genomics, Faculty of Biology, Medicine and Health, School of Biological Sciences University of Manchester Manchester UK; ^2^ Manchester Centre for Genomic Medicine, St Mary's Hospital Manchester University NHS Foundation Trust Manchester UK; ^3^ Present address: Fanxuan Deng Merck Sharp & Dohme China

**Keywords:** ACMG, loss‐of‐function, LZTR1, schwannomatosis, variant classification

## Abstract

Schwannomatosis is a rare tumor predisposition syndrome that causes multiple schwannomas. Germline loss‐of‐function (LoF) *LZTR1* variants were only recently identified as disease‐causing, so relatively few variants have been identified in patients. In addition, many LoF variants exist in Genome Aggregation Database (gnomAD) in people who do not have clinical symptoms of schwannomatosis. These factors, and the incomplete penetrance seen in this condition, hinder definitive interpretation of the clinical significance of novel LoF variants identified in schwannomatosis patients. We collated published LOF *LZTR1* variants identified in schwannomatosis patients and classified them according to current American College of Medical Genetics and Genomics/Association for Molecular Pathology/Association of Clinical Genomic Science guidelines. Subsequently, pathogenic/likely pathogenic schwannomatosis‐associated LoF variants were compared with LoF *LZTR1* variants reported in gnomAD data. Using current classification guidelines, 64/71 LoF *LZTR1* variants reported in schwannomatosis patients in the literature were classified as pathogenic/likely pathogenic, and their frequency in probands 64/359 (17.8%) was significantly higher than the frequency of potential LoF variants identified in the general population (0.36%; *p *< 0.0001). The majority of published classifications of schwannomatosis‐associated LoF variants are robust. However, the high frequency of LoF *LZTR1* variants in the general population suggests that *LZTR1* variants confer a reduced risk of schwannomas compared to germline *NF2* and *SMARCB1* pathogenic variants, making classification of novel variants challenging.

## Graphical abstract

Loss‐of‐function (LoF) LZTR1 variants have been associated with schwannomatosis. However, many LoF LZTR1 variants exist in Genome Aggregation Database (gnomAD) in people who do not have clinical symptoms of schwannomatosis. We show here that LoF LZTR1 variants are strongly enriched in schwannomatosis patients, but the high frequency of LoF LZTR1 variants in gnomAD data suggest a reduced risk of schwannomas in comparison to other schwannoma predisposing genes.

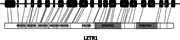

## 1 INTRODUCTION

Schwannomatosis (incl. MIM# 162091 and 615670) is a rare disorder, with an estimated birth incidence of 1 in 57,500–69,000 individuals and a prevalence of 1 in 126,000 individuals (Evans et al., 2018). It is characterized by multiple schwannomas that mainly develop on the spinal and peripheral nerves. Meningiomas and unilateral vestibular schwannomas are rare (Merker et al., 2012; Smith et al., 2017, 2012). About one‐third of patients develop segmental schwannomas, with schwannomas apparently confined to a body segment such as a limb or several spinal nerve roots (Alaidarous et al., 2019). Patients often present with asymptomatic masses and local or systemic neuropathic pain regardless of the location and size of the tumors (Merker et al., 2012). Most patients with schwannomatosis are sporadic, while familial cases account for 13%–25% of all cases. Even within the same family, there is a high clinical variability of schwannomatosis. As an autosomal dominant inherited disorder with incomplete penetrance, many parents or siblings of the proband may carry the pathogenic variant without any clinical symptoms (Kresak & Walsh, 2016).

Schwannomatosis is caused by heterozygous germline pathogenic variants in known predisposing genes *SMARCB1* (MIM# 601607) or *LZTR1* (MIM# 600574) located on chromosome 22q11.2 (Hulsebos et al., 2007; Piotrowski et al., 2014), but other predisposing genes are likely in view of the absence of a germline variant to account for at least 14%–30% of even familial cases.

Germline *SMARCB1* pathogenic variants account for up to 48% of familial cases and 10% of sporadic cases, while the germline *LZTR1* pathogenic variants account for up to 38% of familial cases and 30% of sporadic cases (Boyd et al., 2008; Hadfield et al., 2008; Hutter et al., 2014; Rousseau et al., 2011; Sestini et al., 2008; Smith et al., 2015, 2014, 2012), and up to 80% of schwannomatosis cases with 22q loss in tumors and lacking a pathogenic variant in *SMARCB1 *(Piotrowski et al., 2014). In addition, molecular tumorigenesis mechanisms in both *SMARCB1*‐ and *LZTR1*‐associated schwannomatosis do not conform to the classic Knudson two‐hit model hypothesis, but instead follow a four‐hit/three‐step model that includes loss of heterozygosity (LOH) of the wild‐type allele and somatic biallelic inactivation of the *NF2* (MIM# 607379) gene (Kehrer‐Sawatzki et al., 2017; Sestini et al., 2008).

The genetic characteristics of disease‐associated *SMARCB1* variants have already been studied and genotype–phenotype correlations have been identified between schwannomatosis and other disorders. Most patients with schwannomatosis express germline *SMARCB1* missense variants, in‐frame deletions, or splice‐site variants, mainly located at the 5′‐end or 3′‐end of the gene (Kohashi & Oda, 2017; Smith et al., 2014). In comparison, truncating variants and whole gene, or multiple exons, deletions are related to atypical teratoid/rhabdoid tumors, malignant rhabdoid tumors, and malignant peripheral nerve sheath tumors (Biegel et al., 1999). However, there are still many uncertainties regarding the pathogenicity of schwannomatosis‐associated *LZTR1* variants, which are spread throughout the gene and include missense, nonsense, frameshift, and splice‐site variants (Kehrer‐Sawatzki et al., 2017). Since Piotrowski et al. (2014) identified *LZTR1* variation in schwannomatosis and analyzed the spectrum of pathogenic variants to show that loss‐of‐function (LoF) was the principal disease mechanism, multiple studies have confirmed that LoF variants of *LZTR1* are disease‐causing (Bigenzahn et al., 2018; Hutter et al., 2014; Paganini et al., 2015; Smith et al., 2015; Steklov et al., 2018). However, it is worth noting that many truncating variants and variants located at positions ±1 or 2 (GT‐AG) of canonical splice‐sites, which are considered likely to be pathogenic, also exist in the Genome Aggregation Database (gnomAD) database (https://gnomad.broadinstitute.org/), which represents a large cohort of people who are not known to have clinical symptoms of schwannomatosis. This, along with the incomplete penetrance of schwannomatosis, makes the classification of some predicted pathogenic LoF *LZTR1* variants challenging. The majority of published schwannomatosis‐associated variants were identified before the American College of Medical Genetics and Genomics/Association for Molecular Pathology/Association of Clinical Genomic Science (ACMG/AMP/ACGS) guidelines were widely adopted and were therefore not given a formal classification according to these guidelines in their original publications.

To understand the effect of LoF *LZTR1* variants in schwannomatosis patients further, we compiled a robust cohort of published schwannomatosis‐associated LoF *LZTR1* variants for comparison with *LZTR1* LoF variants seen in the non‐cancer gnomAD dataset. The study involved a literature search of published schwannomatosis‐associated *LZTR1* variants, and classification of those LoF variants based on current ACMG/AMP guidelines (Richards et al., 2015) and ACGS guidelines v4.01 2020 (https://www.acgs.uk.com/quality/best-practice-guidelines/#VariantGuidelines), including updates on the application of cosegregation data (Jarvik & Browning, 2016), ACMG classifier PVS1 (Abou Tayoun et al., 2018) and the scaled points‐based system (Garrett et al., 2021; Tavtigian et al., 2018, 2020). Subsequent data mining of the gnomAD database was used to compare the frequency of published LoF variants identified in people with schwannomatosis to those potential pathogenic variants that have been found in the general population.

## 2 MATERIALS AND METHODS

### 2.1 Literature review for schwannomatosis‐associated *LZTR1* screened patient cohort

To obtain information about LoF *LZTR1* variants among schwannomatosis patients, PubMed was searched using the MeSH words “LZTR1” and “Schwannomatosis.” A total of 49 publications were retrieved. After exclusions, 13 publications were finally selected. The earliest article was published in February 2014, and the most recent was published in January of 2021 (Accessed September 24, 2021).

Patients without a definite clinical diagnosis of schwannomatosis (Evans et al., 2018; Plotkin et al., 2014) were excluded, as well as patients without lymphocyte DNA for sequencing. Patients who met schwannomatosis clinical diagnostic criteria and had undergone molecular testing for *SMARCB1*, *NF2*, and *LZTR1* germline pathogenic variants in peripheral blood DNA through Sanger sequencing or next‐generation sequencing were included. These cases may be unrelated sporadic, or familial. For familial cases, only probands were included in the patient cohort. All 13 publications provided the number of schwannomatosis patients screened as germline *NF2* and *SMARCB1* negative, which was used to calculate the final cohort size.

### 2.2 gnomAD cohort

General population data and the total number of LoF *LZTR1* variants were taken from the gnomAD (Lek et al., 2016). All data were based on Ensembl canonical transcript for *LZTR1* (ENST00000215739.8). The date of download of the data was October 13, 2021. All loss‐of‐function (pLoF) variants in the non‐cancer cohort including nonsense, frameshift variants, and variants located at positions ±1 or 2 (GT‐AG) of canonical splice sites were downloaded. After excluding variants flagged for dubious quality or annotation, the average total number of individuals genotyped in the non‐cancer gnomAD cohort was calculated by summing the “allele number” values for each variant, dividing the sum by the number of different LoF variants, and then dividing by two to account for the two alleles per person (111,103 individuals). Subsequently, variants that were classified as “benign/likely benign” in ClinVar were removed, and the sum of the “allele count” of the filtered variants represented the total number of potential LoF *LZTR1* variants in the general population (396 total variants; 177 different variants).

### 2.3 Maximum credible allele frequency

Using the alleleFrequencyApp (http://cardiodb.org/allelefrequencyapp/) tool developed by Whiffin et al. (2017) along with recommended parameters, to calculate maximum credible population allele frequency, we set the penetrance to 50% (as low as possible, although this is based on 10 of 20 tested relatives of probands in Manchester who had schwannomas). Since *LZTR1* variants account for approximately 30% of schwannomatosis cases, we used 0.3x the calculated birth incidence of 1/57,464 (Evans et al., 2018), that is, 1/191,547, selected monoallelic inheritance, and set allelic heterogeneity to 0.07 (since the most frequent variant in the schwannomatosis cohort, c.27delG, accounted for 5/71 [7%] of variants) and genetic heterogeneity to 1 (as we are only considering *LZTR1*‐associated schwannomatosis), meaning that no single variant causes more than 7% of cases. Using these parameters, the maximum credible population allele frequency of *LZTR1* variants is calculated to be 3.65 × 10^−^^7^ and the maximum tolerated reference allele count in 111,103 individuals is 0. The allele frequency of every LoF *LZTR1* variant found in gnomAD (https://gnomad.broadinstitute.org/transcript/ENST00000215739?dataset=gnomad_r2_1; Accessed October 1, 2021) is greater than 3.65 × 10^−^^7^, suggesting that if a variant is found in gnomAD, it should be considered too common to be causative of schwannomatosis.

### 2.4 Application of evidence classifications

Variants identified in schwannomatosis patients were classified based on the American College of Medical Genetics and Genomics and the Association for Molecular Pathology (ACMG/AMP) guidelines (Richards et al., 2015) and the Association of Clinical Genomic Science (ACGS) guidelines v4.01 2020, including updates on the application of cosegregation data (Jarvik & Browning, 2016), PVS1 classifications (Abou Tayoun et al., 2018), and the scaled points‐based system (Garrett et al., 2021; Tavtigian et al., 2018, 2020). Specific application of ACMG/AMP evidence classifiers in this study is detailed in the Supporting Information Methods and Table S1.

### 2.5 Statistical analysis

The two‐tailed *z*‐score test for two population proportions was used to compare the frequency of *LZTR1* variants in the patient cohort and general population cohort. Pearson's *χ* ^2^ test was used to compare the frequencies of LoF *LZTR1* variants and *SMARCB1* variants in the general population. Odds ratios were calculated using the formula ad/bc, where a = cases with an *LZTR1* LoF variant, b = cases without an *LZTR1* LoF variant, c = controls with an *LZTR1* LoF variant, and d = controls without an *LZTR1* LoF variant.

## 3 RESULTS

### 3.1 Frequency of *LZTR1* LoF variants in schwannomatosis

A total of 359 eligible schwannomatosis patients were included in the study, and germline LoF *LZTR1* variants were detected in 71 cases. Of the 71 variants, 56 were different. The final numbers are shown in Table 1.

**Table 1:** Total schwannomatosis probands screened and *LZTR1*‐positive cases reported in publications

Publications	Total probands screened	Cases with *LZTR1* germline variants
Piotrowski et al. (2014)	20	8
Hutter et al. (2014)	23	5
Paganini et al. (2015)	71	14
Smith et al. (2015)	65	10
Farschtschi et al. (2016)	1	1
Smith et al. (2017)	47	1
Louvrier et al. (2018)	82	16
Kehrer‐Sawatzki et al. (2018)	15	6
Jordan et al. (2018)	22	4
Deiller et al. (2019)	2	2
Alaidarous et al. (2019)	9	2
Herrero San Martin and Alcala‐Galiano (2020)	1	1
Muthusamy et al. (2021)	1	1
Total	359	71

The 56 different *LZTR1* variants were distributed both in introns and almost all exons of the *LZTR1* gene. We assessed the pathogenicity classification of each *LZTR1* variant identified in the patient group. The result of the classification is detailed in Table S1.

A total of 36/56 different variants were absent in gnomAD and two were present but flagged for low confidence or quality in the data. As it was known that LoF of the LZTR1 protein had been associated with human disorders such as schwannomatosis and Noonan syndrome, all nonsense variants and frameshift variants except c.2487dupA (in exon 21, the last exon) were assigned PVS1. Only four splice‐site variants (c.264‐13G>A, c.321‐2delA, c.594‐3C>G, and c.791+1G>A) had messenger RNA analysis data determining truncated transcripts, and all were allocated PVS1. RNA analysis of the +1 splice acceptor variant, c.1449+1G>A identified an in‐frame transcript lacking exon 13 and was assigned PVS1_M. The noncanonical variant, c.652‐34G>T, was not predicted to affect splicing (assigned BP4) and the predictions for c.2220‐16_2220‐14delCTT were conflicting (no classifier assigned).

Although possible cosegregation was observed in many families, there was insufficient data included in the publications to assess criteria, so PP1 was not assigned. BS4 was applied to c.1602delA, which was absent in an affected relative(Paganini et al., 2015); however, this variant still met the criteria to be classified as likely pathogenic overall.

**Figure 1:**
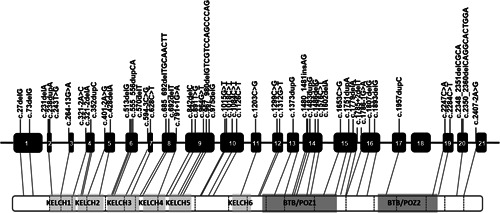
Cartoon of the *LZTR1* gene and protein product, indicating the locations of schwannomatosis‐associated variants that were classified as pathogenic or likely pathogenic in this study.

After categorizing the pathogenicity of 56 different variants in schwannomatosis patients, one variant classified as likely benign and four variants classified as variants of uncertain significance (VUS) were removed from the cohort of *LZTR1*‐LoF variant positive patients, leaving 64 of 71 (49 of 56 different variants), which were included in the comparison with *LZTR1* LoF variants reported in the general population. The gene location of each variant that was classified as pathogenic or likely pathogenic is shown in Figure 1.

### 3.2 Comparing *LZTR1* LoF variants versus *SMARCB1* LoF variants in the general population

There were 177 different *LZTR1* LoF variants (excluding 10 dubious variants and 1 benign/likely benign variant) in the non‐cancer gnomAD cohort, which occurred in 396 alleles in total. The average number of individuals screened for these variants, representing the general population, was 111,103. Therefore, the frequency of LoF *LZTR1* variants in gnomAD was evaluated to be 396 from a mean of 111,103 individuals (1 in 281) including nonsense, frameshift variants, or variants located at positions ±1 or 2 (GT‐AG) of canonical splice‐sites. By contrast, *SMARCB1* only reported three LoF variants in the non‐cancer gnomAD cohort (Ensembl canonical transcript ENST00000263121.7, Accessed February 3, 2022), which means that there were 3 from a mean of 118,374 individuals (1 in 39,458) with an LoF variant. The frequency of LoF *LZTR1* variants in the general population (0.36%) was extremely significantly higher than the frequency of LoF *SMARCB1* variants in the general population (0.0025%) (*p *< 0.0001).

The 396 potential disease‐causing LoF *LZTR1* variants identified in 111,103 individuals in the non‐cancer gnomAD population were compared to the 64 LoF *LZTR1* variants identified in 359 probands (17.8%) that were predicted to be pathogenic in the schwannomatosis cohort. These proportions of LoF variants in the disease cohort and the control cohort were extremely statistically significantly different (*p *< 0.0001). The 64 variants in patients include 21 nonsense variants (32.8%), 32 frameshift variants (50%) and 11 splice‐site variants (17.2%), while the 396 variants in the general population include 165 nonsense variants (41.7%), 120 frameshift variants (30.3%), and 111 splice‐site variants (28.0%). Subsequently, the frequencies of the three different types of variants were compared separately between the schwannomatosis population and the general population (Table 2) and the proportions were also extremely significantly different.

**Table 2:** Comparison of the frequency of loss‐of‐function *LZTR1* variants between Schwannomatosis patients and the general population

Variant types	Reported in patients	Reported in controls	Odds ratio^a^
Nonsense variants	21/359 (5.8%)	165/111,103^b ^(0.15%)	41.8
Frameshift variants	32/359 (8.9%)	120/111,103^b^ (0.11%)	90.5
Splice‐site variants	11/359 (3.1%)	111/111,103^b^ (0.10%)	31.6

*Note*: All *p* < 0.0001.

^a^ Odds ratios were calculated using ad/bc, where a = cases with an *LZTR1* loss‐of‐function (LoF) variant, b = cases without an *LZTR1* LoF variant, c = controls with an *LZTR1* LoF variant, and d = controls without an *LZTR1* LoF variant.

^b^ Average number of individuals tested.

Since the use of highly selected cohorts in the published literature before *LZTR1* screening, for example, by known 22q involvement in tumors and/or exclusion of *SMARCB1* positive cases may produce an inaccurate denominator, we calculated the proportion of *LZTR1* LoF variants in our own current total of 359 isolated (nonfamilial) cases tested in Manchester, not selected for 22q involvement and which include patients who tested positive for a *SMARCB1* pathogenic variant. In this cohort, there were 61 people with an LoF *LZTR1* variant (17%), which is very similar to the proportion of *LZTR1* LoF positive people in the combined literature used in the current study.

### 3.3 Likelihood of schwannomatosis in *LZTR1* LoF carriers in the general population

Since the birth incidence of schwannomatosis is 1/57,464 (Evans et al., 2018) and we have shown here that 17% of these have a germline pathogenic or likely pathogenic LoF *LZTR1* variant i.e. 1/338,024, and since we have shown that 1/281 people in the non‐cancer gnomAD population have a predicted pathogenic or likely pathogenic *LZTR1* variant, this means that 1 in 1203 (338,024/281) people in the general population carrying a potential pathogenic *LZTR1* variant would be expected to develop clinical schwannomatosis. Since this frequency of *LZTR1*‐associated schwannomatosis (1/1203) is not observed this suggested that *LZTR1* carries a lower risk of disease than a typical autosomal dominant gene.

The most common single LoF variant in people with schwannomatosis was c.27delG, p.(Gln10ArgfsTer15), which was seen in 5/359 probands with schwannomatosis and was seen in 10/77,768 people on gnomAD, giving an odds ratio of 110. The second most common LoF variant identified in schwannomatosis probands was c.264‐13G>A, which was seen in 4/359 people with schwannomatosis, and 10/118,437 people, giving an odds ratio of 133.4. These frequencies indicate a strong association of *LZTR1* with a schwannomatosis phenotype, supporting our use of PS4_M for these variants.

### 3.4 Likelihood of false positives

In an isolated case meeting schwannomatosis criteria the odds ratio for finding an apparently causative *LZTR1* variant is 1:5.88 against (i.e., 17%). This compares to the likelihood of finding a variant by chance of 1 in 281. As such the chances of finding a false positive could be 1 in 48 (2.1%). This would increase further if there was no loss of the wild‐type allele in tumor DNA (close to 100%). We have analyzed 40 schwannomas from people with an apparently pathogenic/likely pathogenic germline *LZTR1* variant and 36 (86%) also have LOH of the wild‐type allele. This compares to 22/22 (100%) tumors from people with a germline pathogenic *SMARCB1* variant. Since LOH indicates an obligate pathway to schwannoma mediated by *LZTR1*/*SMARCB1*, this suggests that further potentially pathogenic *LZTR1* variants may not be the cause of schwannomatosis and that additional tumor data are needed to confirm the causality of a novel *LZTR1* variant found in a schwannomatosis patient.

## 4 DISCUSSION

The results of clinical genetic testing play a significant role in the management of hereditary tumors, which can often provide pivotal information for screening, diagnosis, surgical recommendations, and treatment decisions (Garrett et al., 2021). We have assessed the current evidence available for published LoF *LZTR1* variants identified in schwannomatosis patients, many of which were published before the widespread adoption of ACMG/AMP/ACGS classification guidelines. We also addressed some of the confounding factors that make the classification of *LZTR1* variants particularly challenging.

### 4.1 Published schwannomatosis‐associated *LZTR1* variants

From the results of our assessment of published schwannomatosis‐associated variants using ACMG/AMP/ACGS guidelines, all schwannomatosis‐associated nonsense and frameshift variants met the criteria to be interpreted as pathogenic or likely pathogenic, except c.2487dupA, which occurs in the final exon. However, six splice‐site variants were classified as VUS based on available published data. It is possible that these variants could be reclassified as pathogenic/likely pathogenic with appropriate family history data and functional studies. There is not currently enough evidence on the effects of specific splice variants that disrupt or remove particular functional LZTR1 protein domains to use this information with confidence. In addition, transcripts are generally predicted to escape NMD when the premature termination codon occurs in the last exon or the last 50 nucleotides of the penultimate exon. However, it is not clear whether this prediction is also true for *LZTR1*. Therefore, not only in silico analysis but also in vivo/in vitro studies for candidate variants as well as investigation of the family history will all be critical for future variant interpretation.

### 4.2 Maximum allele frequency

Classification of pathogenicity of variants is important and challenging work. There is no doubt that the development of the ACMG/AMP/ACGS guidelines has greatly improved the consistency of the interpretation of variants among different institutions or individuals. However, the process of implementing classification guidelines is still subjective, which is also a limitation of this study. This is reflected in the application of frequency and population data. Accurate and extensive epidemiological and population genetic studies are required to calculate an accurate maximum credible population allele frequency. However, there are few reports about schwannomatosis population frequency and disease penetrance. Therefore, the credible allele frequency limit of 3.65 × 10^−^^7^ cannot be applied to classification with great confidence. Although the largest allele frequency of LoF variants (p.Trp469*, 0.015%) is lower than the cutoff of 0.1%, which usually represents a rare variant, this frequency may still be considered too high compared to the birth incidence of schwannomatosis of (0.0017%). In fact, 20 different variants in the patient cohort (including two flagged for low confidence) had been seen in the gnomAD population. This finding, and the incomplete penetrance of disease seen in *LZTR1*‐associated schwannomatosis, suggests that germline *LZTR1* variants may carry a lower risk of symptomatic schwannoma disease than other genes, such as *SMARCB1* and *NF2*. However, an intriguing aspect of *LZTR1*‐related schwannomatosis is that despite recorded nonpenetrance in schwannomatosis families with an apparent pathogenic/likely pathogenic variant a relatively high number of relatives do develop schwannomas (50% in Manchester). The mere fact that 38% of “familial” schwannomatosis families have an *LZTR1* variant means that there has to be more than one clinically affected case in each of those families. It is, therefore, possible that a genetic factor in close linkage with *LZTR1* is required to activate schwannoma formation when an *LZTR1* LoF variant is present. This might explain the relatively high penetrance in known families, but low penetrance is assumed based on gnomAD frequencies. This means that when LoF *LZTR1* variants are detected in patients, more evidence is needed clinically to determine if they are part of the etiology of schwannomatosis.

Missense variants have also been associated with schwannomatosis; however, in this study, we have focused on LoF variants, as missense variants are particularly difficult to classify. Another complicating factor is that, similar to *SMARCB1*, *LZTR1* has also been associated with other genetic conditions(Frattini et al., 2013; Yamamoto et al., 2015). In particular, there is an overlap between the *LZTR1* variants seen in schwannomatosis and those seen in rare recessive forms of *LZTR1*‐associated Noonan syndrome (Johnston et al., 2018), which is not a tumor predisposing condition. The mechanism of pathogenicity is less clear in Noonan syndrome and further research is needed to determine the role of these variants in different disorders.

### 4.3 Family segregation

Family history information always plays a significant role in variant interpretation. Testing the relatives to determine whether the variant identified in the proband is *de novo*, or whether there is sufficient co‐segregation with disease phenotype within a schwannomatosis family, increases the confidence in the interpretation of a novel variant. However, the extensive incomplete penetrance observed in schwannomatosis affects the utilization of family history information and may also make it difficult to distinguish sporadic cases from familial cases. A lack of correlation of an *LZTR1* variant with the disease within a family may be due to the following reasons: (1) The *LZTR1* variant detected in the proband is not pathogenic, (2) the *LZTR1* variant may lead to a mild phenotype, making individuals with schwannomatosis mild or asymptomatic clinically, and (3) since the mean age of onset of schwannomatosis is around 40 years old (Evans et al., 2018), carriers may be too young to have developed symptoms. (4) Due to a lack of full‐body magnetic resonance imaging examination, some tumors remain undetected. (5) An unidentified additional modifier effect (Kehrer‐Sawatzki et al., 2017). As the cohort of reported patients with schwannomatosis expands, more unaffected carriers in the family will be discovered and these issues may be addressed by analyzing the genotype, age, imaging reports, and tumor data of these individuals on a large scale.

One reason that the allele frequency of some *LZTR1* variants identified in many patients is higher than the expected value in the general population may be that the prevalence of *LZTR1*‐associated schwannomatosis is higher than previously thought. In future research, an assessment of the proportion of asymptomatic carriers of germline *LZTR1* variants in relatives of affected individuals would be highly valuable.

### 4.4 Tumor data

Additionally, evidence of pathogenicity for schwannomatosis‐associated *LZTR1* variants should not rely solely on interpreting the germline *LZTR1* variants, but also on analysis of the tumor DNA when available.

A previous report of seven schwannomatosis patients carrying a germline *LZTR1* variant, determined that two of these people (one carrying a nonsense *LZTR1* variant and the other carrying a missense *LZTR1* variant) also had identical *NF2* pathogenic variants in two anatomically distinct tumors, indicating mosaic NF2 (Kehrer‐Sawatzki et al., 2018). To be certain of the association of LZTR1 with a schwannomatosis phenotype, the same *LZTR1* variant as found in peripheral blood should be detected in at least two tumor samples, along with LOH of the wild‐type allele, and different somatic *NF2* single nucleotide variants should be detected in each tumor sample. This situation will significantly increase the possibility that the germline variant is causative. However, in clinical practice, tumor samples are not easy to obtain because many schwannomatosis patients do not undergo surgery. Not surprisingly, in the publications included in this study, many patients did not have tumor information available, and fewer patients had two tumor samples available. The addition of tumor‐related evidence will be an important part of future schwannomatosis‐specific variant classification guidelines.

## 5 CONCLUSION

Like any other rare disease, one issue that hinders interpretation of the pathogenicity of *LZTR1* variants detected in clinical laboratories is there are so few schwannomatosis cases, leading to an insufficient level of evidence. Some general information‐sharing databases have already been established on a global scale such as ClinVar, but they do not always include enough evidence to support their classification. Therefore, it will be important to develop schwannomatosis‐specific databases, such as the international schwannomatosis registry (Ostrow et al., 2017), where institutes worldwide can upload phenotypes, genotypes, tumor information, functional and predictive data, and available family history so as to promote in‐depth research on schwannomatosis susceptibility genes and to aid confident classification of novel risk variants. In addition, it will be important to develop gene‐specific classification guidelines. A neurofibromatosis and schwannomatosis‐specific ClinGen variant curation expert panel has been formed recently to develop these gene‐specific guidelines and we have highlighted areas that will need to be addressed by the panel within *LZTR1*‐schwannomatosis in the first iteration of these guidelines.

## AUTHOR CONTRIBUTIONS

**Miriam J. Smith and D. Gareth Evansr**: Conceptualization. **Fanxuan Deng, D. Gareth Evans, and Miriam J. Smith**: Formal analysis. **Fanxuan Deng and Miriam J. Smith**: Writing – original draft. **D. Gareth Evans and Miriam J. Smith**: Writing – review and editing.

## CONFLICTS OF INTEREST

The authors declare no conflicts of interest.

## WEB RESOURCES

gnomAD: https://gnomad.broadinstitute.org/; Ensembl: https://www.ensembl.org/index.html; ACGS guidelines v4.01 2020: https://www.acgs.uk.com/quality/best-practice-guidelines/#VariantGuidelines; Allele Frequency Filter: http://cardiodb.org/allelefrequencyapp/; SpliceAI https://spliceailookup.broadinstitute.org/; Mutalyzer: https://mutalyzer.nl/; and ClinVar: https://search.clinicalgenome.org/

## Supporting information

Supporting information.

Supporting information.

## Data Availability

The manuscript is based on previously published data that is already available in the published literature.
