# Supplementary material for: Comparison of the frequency of loss‐of‐function LZTR1 variants between schwannomatosis patients and the general population
Source: Hum Mutat. 2022 Apr 14;43(7):919–27. doi: 10.1002/humu.24376 (PMC9324957; doi:10.1002/humu.24376)
Supplement: Supplementary file 1 — Supporting information. [file HUMU-43-919-s001.pdf]

Supplementary Methods S1

Sources of classification guidelines

This study classified variants identified in schwannomatosis patients based on ACMG/AMP guidelines (Richards et al., 2015), including updated guidance from ACGS guidelines v4.01 2020 [https://www.acgs.uk.com/quality/best-practice-guidelines/#VariantGuidelines], and including updates on the application of cosegregation data by Jarvik and Browning (Jarvik and Browning, 2016), application notes on the use of PVS1 classifications by Abou Tayoun et al.(Abou Tayoun et al., 2018), and the scaled points-based system described by Tavtigian et al., (Tavtigian et al., 2018; Tavtigian et al., 2020), and additional points-based combinations described by Garrett et al.(Garrett et al., 2021). Points are used to indicate strength of evidence categories supporting a variant as being either pathogenic (+) or benign (-). Indeterminate evidence gives no points, Supporting evidence gives ±1 point, Moderate evidence gives ±2 points, Strong evidence gives ±4 points and Very Strong evidence gives ±8 points. Points indicate benign variants at <-6, Likely Benign at -1 to -6, VUS at 0 to 5, Likely Pathogenic at 6 to 9 and Pathogenic at 10 points or more (https://www.acgs.uk.com/quality/best-practice-guidelines/#VariantGuidelines).

The temperature scale for variants of uncertain significance suggests 0 points for an ice cold VUS, 1 point for a cold VUS, 2 points for a cool VUS, 3 points for a tepid VUS, 4 points for a warm VUS, and 5 points for a hot VUS (https://www.acgs.uk.com/quality/best-practice-guidelines/#VariantGuidelines).

ACMG classifiers used in this study

Clinical data

When investigating the clinical data of the variant, the principal considerations involve whether the variant is *de novo*, whether the variant segregates with disease in families, and whether the phenotype of the patient is highly specific for schwannomatosis.

ACMG classifier PP4 was applied extensively, since all variants were identified in highly characterized patients meeting schwannomatosis clinical diagnostic criteria.

PS2/PM6 were not used, as although many cases were listed as *de novo*, there was insufficient evidence to determine whether parents had been tested for the variant, and/or whether paternity/paternity had been confirmed.

PP1 was also not used, as there were an insufficient number of meioses to support this according to the Jarvik and Browning (2016) thresholds. We did not apply BS3 in cases where a variant was found in an unaffected individual, due to the known incomplete penetrance of disease in schwannomatosis and age-related appearance of symptoms. However, for the variant which was not seen in an affected relative, BS4 was applied.

Functional data and predictive data

Using recommendations suggested by Abou Tayoun et al., 2018, we assigned the ACMG classifier PVS1 for nonsense variants and frameshift variants that were not in the last exon of the gene, and which were predicted to cause nonsense mediated decay (NMD). Nonsense or frameshift variants in the last exon were assigned PVS1_M. Variants occurring at ±1 or 2 canonical splice-sites, except for the last exon and predicted to skip an out-of-frame exon were also classified as PVS1. For variants occurring at the ±1 or 2 canonical splice-site at the last exon or predicted to skip an in-frame exon, we assigned PVS1_M. For non-canonical splice variants, when RNA analysis proved that the variant caused an out-of-frame truncated transcript, PVS1 was applied. When RNA analysis proved that the variant caused an in-frame truncated transcript, PVS1_M was applied. There is not currently enough data on the effects of specific splice variants that disrupt or remove particular functional LZTR1 protein domains to use this information with confidence. Therefore our assessments are only based on the maintenance or disruption of the overall reading frame of the LZTR1 transcript.

Computational and predictive tools were used to perform *in silico* analysis for splice-site variants to determine the application of PP3 or BP4. For *in silico* predictions we used AlamutVisualPlus, which includes the NNSplice and GeneSplicer algorithms recommended by the ACGS guidelines and SpliceAI (https://spliceailookup.broadinstitute.org/), (Jaganathan et al., 2019) which has been shown to be one of the best performing meta-predictors. Only Splice AI prediction scores >0.5 (indicating a confident prediction of disruption to splicing) were considered to be supportive of splice changes.

Alamut Visual Plus_1.4 reports were also used to assess aberrant splicing, as they include MaxEntScan and SpliceSiteFinder-Like predictions that are recommended by ACGS (Ellard et al. 2020), as well as NNSplice and GeneSplicer, suggested by ACMG (Richards et al. 2015). Where the results were inconclusive no classifier was used, where only *in silico* predictions were available PP3 was used, and the PS3 classifier was not applied in combination with any level of PVS1 classifier.

Variant population frequency

PM2 was applied if *LZTR1* variants had not been reported in gnomAD. Due to the rarity of schwannomatosis disease and the known incomplete penetrance of disease, classifiers BS1 and PS4 were used with a reduced strength. If a variant was seen in 1-2 affected probands and controls, or 2 affected probands and >2 controls, then neither classifier was used. If a variant was seen once in the case cohort and >2 times in controls, then BS1 was used at a moderate level (BS1_M). If a variant was seen in >2 times in the case cohort and >2 times in controls, then PS4 was used at a moderate level (PS4_M).

References

Abou Tayoun, A. N., Pesaran, T., DiStefano, M. T., Oza, A., Rehm, H. L., Biesecker, L. G., Harrison, S. M., ClinGen Sequence Variant Interpretation Working, G. (2018). Recommendations for interpreting the loss of function PVS1 ACMG/AMP variant criterion. *Hum Mutat* 39(11):1517-1524.

Garrett, A., Durkie, M., Callaway, A., Burghel, G. J., Robinson, R., Drummond, J., Torr, B., Cubuk, C., Berry, I. R., Wallace, A. J., Ellard, S., Eccles, D. M. et al. (2021). Combining evidence for and against pathogenicity for variants in cancer susceptibility genes: CanVIG-UK consensus recommendations. *J Med Genet* 58(5):297-304.

Jaganathan, K., Kyriazopoulou Panagiotopoulou, S., McRae, J. F., Darbandi, S. F., Knowles, D., Li, Y. I., Kosmicki, J. A., Arbelaez, J., Cui, W., Schwartz, G. B., Chow, E. D., Kanterakis, E. et al. (2019). Predicting Splicing from Primary Sequence with Deep Learning. *Cell* 176(3):535-548 e24.

Jarvik, G. P., Browning, B. L. (2016). Consideration of Cosegregation in the Pathogenicity Classification of Genomic Variants. *Am J Hum Genet* 98(6):1077-1081.

Richards, S., Aziz, N., Bale, S., Bick, D., Das, S., Gastier-Foster, J., Grody, W. W., Hegde, M., Lyon, E., Spector, E., Voelkerding, K., Rehm, H. L. et al. (2015). Standards and guidelines for the interpretation of sequence variants: a joint consensus recommendation of the American College of Medical Genetics and Genomics and the Association for Molecular Pathology. *Genet Med* 17(5):405-24.

Tavtigian, S. V., Greenblatt, M. S., Harrison, S. M., Nussbaum, R. L., Prabhu, S. A., Boucher, K. M., Biesecker, L. G., ClinGen Sequence Variant Interpretation Working, G. (2018). Modeling the ACMG/AMP variant classification guidelines as a Bayesian classification framework. *Genet Med* 20(9):1054-1060.

Tavtigian, S. V., Harrison, S. M., Boucher, K. M., Biesecker, L. G. (2020). Fitting a naturally scaled point system to the ACMG/AMP variant classification guidelines. *Hum Mutat* 41(10):1734-1737.
